# Functional Analysis beyond Enrichment: Non-Redundant Reciprocal Linkage of Genes and Biological Terms

**DOI:** 10.1371/journal.pone.0024289

**Published:** 2011-09-16

**Authors:** Celia Fontanillo, Ruben Nogales-Cadenas, Alberto Pascual-Montano, Javier De Las Rivas

**Affiliations:** 1 Cancer Research Center (CiC-IBMCC, CSIC/USAL), Campus Miguel de Unamuno, Salamanca, Spain; 2 National Center of Biotechnology (CNB, CSIC), Campus de Cantoblanco UAM, Madrid, Spain; Rutgers University, United States of America

## Abstract

Functional analysis of large sets of genes and proteins is becoming more and more necessary with the increase of experimental biomolecular data at *omic*-scale. Enrichment analysis is by far the most popular available methodology to derive functional implications of sets of cooperating genes. The problem with these techniques relies in the redundancy of resulting information, that in most cases generate lots of trivial results with high risk to mask the reality of key biological events. We present and describe a computational method, called ***GeneTerm Linker***, that filters and links enriched output data identifying sets of associated genes and terms, producing metagroups of coherent biological significance. The method uses fuzzy reciprocal linkage between genes and terms to unravel their functional convergence and associations. The algorithm is tested with a small set of well known interacting proteins from yeast and with a large collection of reference sets from three heterogeneous resources: multiprotein complexes (CORUM), cellular pathways (SGD) and human diseases (OMIM). Statistical *Precision*, *Recall* and balanced *F-score* are calculated showing robust results, even when different levels of random noise are included in the test sets. Although we could not find an equivalent method, we present a comparative analysis with a widely used method that combines enrichment and functional annotation clustering. A web application to use the method here proposed is provided at http://gtlinker.cnb.csic.es.

## Introduction

Genome- and proteome-wide analyses performed using high-throughput techniques are providing many collections of genes and proteins that are associated to studies performed over specific sets of samples in definite biological contexts. One of the major challenges of current computational biology is to provide robust automatic methods for a meaningful functional annotation of the long lists of genes or proteins derived from such high-throughput studies. Functional *enrichment analysis* (EA) is at present the most popular available methodology to derive functional implications of sets of cooperating genes. It uses statistical testing to find significant annotations in groups of genes. A recent review of enrichment tools categorizes them in three major classes: *singular* (SEA), *modular* (MEA) and *gene-set* (GSEA) [1]. Modular analysis (MEA) can be considered a second generation of functional enrichment since it uses concurrent gene annotation improving coverage [2], [3], [4]. Gene set enrichment analysis (GSEA) has become a popular tool to extract biological insight from complete ranked gene lists without the need of pre-selecting top genes [5].

Functional enrichment analysis, however, does not address several key problems associated to the biological annotations: ***(i)*** *Redundancy* of the biological terms, that are repeated in many different annotation resources (e.g. *cell cycle* GO:0007049, *cell cycle* KEGG hsa04110, etc) or that are segregated in very similar terms with the same biological meaning (e.g. GO:0007049 *cell cycle* and GO:0022402 *cell cycle process*). ***(ii)*** *Bias* in the annotation space due to highly frequent use of certain “promiscuous” terms that are unspecific (e.g. GO:0050789 *regulation of biological process* includes more than 44% of all human genes annotated to GO-BP). ***(iii)*** *Inadequate functional annotation* of many genes that are well-known (e.g. NRAS human gene product P01111 is not annotated to GO:0043410 *positive regulation of MAPKKK cascade*, but the role of this gene in the MAPK signaling is well-known, since it is paralogous to gene HRAS, which has a central role in such pathway).

To overcome these limitations and challenges we have developed a new computational method that finds significant and coherent metagroups of genes and terms, performing several steps to eliminate redundant and non-informative data. The method takes the output of an enrichment analysis and produces a simple result that includes genes and co-annotations associated in metagroups. These metagroups are ranked by analysis of their significance and coherence, as a way to find the most relevant functions present in the query gene list. The algorithm is tested with a small set of well known interacting proteins and with a large reference set of data from three heterogeneous resources: mammalian multiprotein complexes (CORUM), yeast cellular pathways (SGD) and human diseases (OMIM). Statistical *Precision*, *Recall* and balanced *F-score* are calculated for each test, and we observe robust results even introducing different percentages of randomly selected genes in the queries. The computational method can be applied to the output result of any enrichment analysis. We provide a web application to use the method (http://gtlinker.cnb.csic.es) that only needs as input a gene list, because in a first step it runs an enrichment analysis tool [3] implemented within the same workflow.

## Results

### Analysis of the distributions of terms/genes in different Annotation Spaces

Functional annotation and enrichment analysis relies on the use of biological databases that include groups of genes associated to specific biological functions, such as: metabolic and signaling pathways, cellular processes and apparatus, organisms, etc. Some of the biological databases most used in functional profiling are: GO (repository of gene and gene product ontological attributes across species) [6], KEGG (atlas of biological pathways) [7], UniProt (catalog of structural and functional information on proteins) [8]. In these databases the functions are annotated with specific terms that define and describe the biological roles and actions. They usually apply controlled vocabularies, i. e. structured collections of terms with numerical IDs. As it happens in language evolution, the use of the terms can modulate their meaning, because when some expressions become too trendy, fashionable or promiscuous they can lose significance. In addition, most of these vocabularies are defined to be organism-independent and therefore in some cases they encode global definitions that are not useful to explain very specific biological processes.

**Figure 1:**
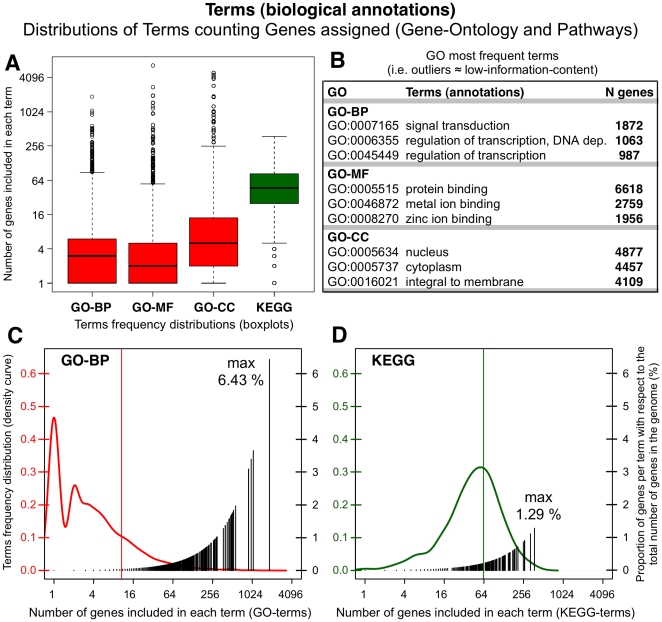
Distributions of biological terms in GO and KEGG databases. Distributions of biological terms from GO and KEGG databases counting the number of genes assigned to each term. The data correspond to human genes. *(A)* Boxplots of the distributions for GO categories (BP, MF, CC) and for KEGG. *(B)* Most frequent GO terms. *(C)* Left: density distribution of GO-BP -marking the average with a red line-; right: proportion of genes per term with respect to the total number of genes (%). *(D)* Same as C for KEGG.

We have analyzed and compared the frequency distributions of the biological terms in two worldwide used databases (GO and KEGG). This analysis counts the number of genes assigned to each term and reveals that the distributions are quite uneven, existing a large proportion of terms that include very small number of genes and a considerable amount of outliers assigned to many genes. In fact, for the case of GO-BP (Biological Process), GO-MF (Molecular Function) and GO-CC (Cellular Component) more than 50% of the terms have less than four genes assigned in human (see **Figure 1A**, boxplots of the distributions of GO and KEGG terms assigned to human genes). The distribution is more homogeneous for the case of KEGG terms, which shows a Gaussian-like curve (**Figure 1C and 1D**). The black vertical lines in these plots indicate the percentage of genes per term with respect to the total number of human genes (i.e. 29095 genes using ENSEMBL v57, March 2010). The results show that the most used GO-BP term is assigned to 6.43% human genes (1872 genes assigned to *signal transduction*, GO:0007165). **Figure 1B** presents for each GO category (BP, MF, CC) the three terms most frequently annotated to human genes. Such terms (e.g. term *protein binding*) are outliers in the distributions (**Figure 1A**) and therefore they can be considered terms with low-information-content, too generic to provide clear and meaningful functional annotation on their own.

### Identification of over-represented terms to improve functional annotation

The analysis of the distribution of terms indicates that there are some biological annotations that are over-represented, mainly in GO. Such over-representation can be quantified by the deviation from the average number of assignments (red and green vertical lines in **Figure 1C and 1D**). Based on such average (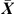) and on the standard deviation (σ_x_) of the distributions of terms in each annotation space for each organism, we set up a *Z-score* threshold to identify the outlier terms that had a number of genes assigned (***Ng***) deviated from average: ***Ng>(*** 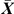 **+nσ_x_)**. The deviation factor ***n*** was set up at 4 for human. This threshold allows identification of the biological terms that are “generic” and “promiscuous”, and –on their own– they can be considered not very informative. These generic terms affect a significant proportion of genes. In the case of human, generic GO-BPs include 10,038 genes (34.5% of the total), generic GO-MFs include 12,991 genes (44.6% of the total) and generic GO-CCs include 15,179 genes (52.2% of the total). In the case of KEGG only 2 terms were considered nonspecific and they only affect to 700 genes. All the generic terms were tagged in order to further use them only in the case that they appear in co-occurrence with other terms.

### Definition of *GeneTerm-sets* as a type of *Frequent Itemsets*

Most of the enrichment analyses are based in searching for *frequent patterns* of association between biomolecular elements (e.g. genes, proteins) and the corresponding annotations or descriptions found in biological databases. In the data-mining field those patterns are called *frequent itemsets* [9]. A formal mathematical definition of *frequent itemsets* can be as follows: given a set of *items* 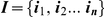 and a database of *transactions* 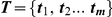 where each transaction is a subset of 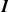, 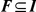 is a *frequent itemset* if it is included in a number of transactions greater than a specified threshold, 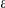. That number of transactions is called the *support* of the itemset.

Translating these concepts to the biological context of enrichment analyses, the *items* will be the “terms” (i.e. the biological annotations) from the different databases, and the *transactions* will be the “genes” (i.e. the biological entities). In this way, it is possible to generalize the *frequent patterns* obtained by any enrichment analysis as a list of annotations related with a subset of genes, both associated by the score or ***p-value*** of the enrichment that measures the strength of the relationship. Formally, such combination of terms/genes/***p-value*** is a *frequent itemset* derived from a functional annotation procedure, and we define such as *GeneTerm-set* element: 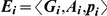. Where ***E_i_*** is the *ith* element in the results, ***A_i_*** is a set 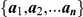 of biological annotations or terms, ***G_i_*** is a set of genes 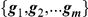 and ***p_i_*** the ***p-value***. In terms of enrichment ***A_i_*** is a set of annotations over-represented in a list of genes and ***G_i_*** is the subset of genes that support that over-representation with a ***p-value*** of ***p_i_***. When using singular (SEA) or concurrent modular (MEA) enrichment analyses, the difference in the data structure of the result consists only in the number of elements in ***A_i_***, that is 1 in the first case and ≥1 in the latest. Most of the enrichment tools provide large lists of these *GeneTerm-set* elements derived from the analysis on different annotation spaces. Such multiple lists are many times very redundant, provided as independent or non-related and including many generic terms. This hampers the extraction of meaningful biological insights because the interpretation of such redundant and complex data sets is quite difficult, time-consuming and daunting, many times dependent on the expertise and the area of interest of the biologists that analyze the lists.

### Method: non-redundant reciprocal linkage of *GeneTerm-sets* to go beyond Enrichment

We have developed a computational method to find metagroups of genes and annotations composed by linked *GeneTerm-sets*, eliminating redundant and non-informative elements. The method, called ***GeneTerm Linker*** has 2 major goals: ***(i)*** to provide a robust automatic way to analyse the large collections of *GeneTerm-sets* produced by enrichment methods; ***(ii)*** to produce significant and coherent metagroups of genes associated to concurrent terms and annotations that describe the specific biological functions of the metagroup. In the following paragraphs we describe the four major procedure steps that the method includes:

### Step 1

#### Filtering *GeneTerm-sets* that only include over-represented terms

As we showed above, those terms whose frequency of appearance in databases is strongly greater than average can provide obvious and non-interesting results, while masking significant functional patterns present in the query genes. Such over-represented terms are considered outliers. Once the outliers are found in each biological annotation category for each organism, the first step of the method consists in removing the *GeneTerm-set* elements that only correlate groups of genes with over-represented terms. If one element in the enrichment result includes outliers in its set of annotations but also contains other terms, the element is not discarded because the generic terms are related with other specific annotations. In this way, given an element ***E_i_*** from the enrichment result, the whole element will be set aside only if its set of annotations ***A_i_*** is composed by outliers. This first step of the method significantly reduces the number of elements in the list of results, removing useless information.

### Step 2

#### Retrieve metagroups using reciprocal linkage between *GeneTerm-sets*

The second step of the algorithm creates metagroups of elements that are related by sharing common genes or by sharing common terms. The method is reciprocal because it considers both the genes and the terms included in each *GeneTerm-set*. First, to find the linkage between genes it uses a similarity coefficient that provides a preliminary grouping of *GeneTerm-sets*. Second, to find the linkage between terms it uses a greedy algorithm that explores the annotations to merge the common ones.

Gupta *et al.* showed that the use of the *Jaccard Similarity coefficient* to measure the distance between the transactions that support frequent patterns get better results than the distance between the items, demonstrating its fitness to catch the interactions between those sets in the data and its robustness regardless the size of the data [10]. This is an approach that does not take into account the strength of the relationships between transactions and items, i.e. between genes and terms in our case. Considering these ideas, our method finds the linkage between *GeneTerm-set* elements by creating for each ***E_i_*** a vector ***v_i_*** which contains the occurrence of each gene with respect to the whole gene list of the input (in binary numbers 1/0) and incorporates as an additional component the ***p-value*** of each element ***E_i_*** weighted by factor ***M*** (the total number of genes in the list). This additional parameter represents the strength of the relationship within each *GeneTerm-set*. The pair-wise distances between all vectors ***v_i_*** are calculated using *Cosine Similarity*, a generalization of the *Jaccard Similarity coefficient* for non-binary attributes. Once the similarity is calculated, the distances are analyzed using *Ward's hierarchical clustering* in order to find the linkage between *GeneTerm-sets* (i.e. the clusters formed by the elements). This linkage is considered fuzzy because each gene or combination of genes can be included in several *GeneTerm-sets*. A heuristic threshold consisting of a cutoff set up at a given depth of the cluster tree is used to define the preliminary metagroups. By default the threshold is set up at 20% of the tree depth, but if it is not enough to define metagroups, the algorithm increases the cutoff in 10% steps till at least one metagroup is found. In this way, we identify coherent modules of information based on common genes.

After this process, the algorithm proceeds performing a greedy recursive exploration of terms within the preliminary clusters (pre-metagroups) to merge the ones that share the same terms. At the end of this second step the method provides metagroups where the convergence of genes and terms is maximized. A formal mathematical description of the process is included in the Materials and Methods.

### Step 3

#### Remove redundancy within the selected metagroups

Once the metagroups are created, it is possible to compact and reduce their size by removing the redundant elements included inside each metagroup.

Toivonen *et al.* proposed the concept of *cover* of a set of association rules (a special case of *frequent itemsets*) as the minimal subset that contains all the relationships present in an original set [11]. To avoid losing any item, we extend the concept of *cover* of a collection of *itemsets* (i.e., in our case, a metagroup of *GeneTerm-sets*) with the requirement of *completeness* of the data. In this way, in our algorithm we redefine and apply the concept of *complete cover*. The mathematical description to calculate this parameter is presented in Materials and Methods.

To assess the *complete cover* we do not contemplate only the terms included in the metagroups, but also the genes that support them. Each metagroup is described by the total set of terms and the total set of genes included in their elements. So, to find redundant elements inside a metagroup the method searches for the ones with all its genes and terms included in another elements of the same metagroup. In this search the *GeneTerm-sets* are always ordered by increasing ***p-values*** to eliminate consistently the less significant sets. Following this approach, redundant *GeneTerm-sets* present in the enrichment outputs are found and removed.

### Step 4

#### Calculate significance and coherence of the metagroups

After the final metagroups have been generated and the redundant *GeneTerm-sets* removed, a series of parameters are calculated to evaluate their significance and coherence. Our assumption is that a functional coherent metagroup should be compact and well separated from other, therefore such coherence tries to measure both the intra-groups compactness and the inter-groups distance.

In order to evaluate the statistical significance a *Hypergeometric test* is performed with all the genes and terms assigned to each metagroup [2], [12]. The resultant ***p-values*** are adjusted for multiple tests using the FDR method [13].

In order to assess the compactness (maximum distance in between data points of clusters) and proximity (minimum distance between clusters) the main parameter calculated is the *Silhouette Width*, which ranges from 1 to −1 and measures both the compactness and proximity of multiple groups [14]. The method also calculates the *Diameter*, that is the maximum *Cosine distance* within the *GeneTerm-sets* of each metagroup and ranges from 0 to 1; and the *Similarity Coefficient*, which is [1 – average *Cosine distance*] within the *GeneTerm-sets* of each metagroup and also ranges from 0 to 1. All these distance and similarity calculations are done based on the genes present in the metagroups.

### Testing the method with a set of yeast nuclear proteins

**Figure 2:**
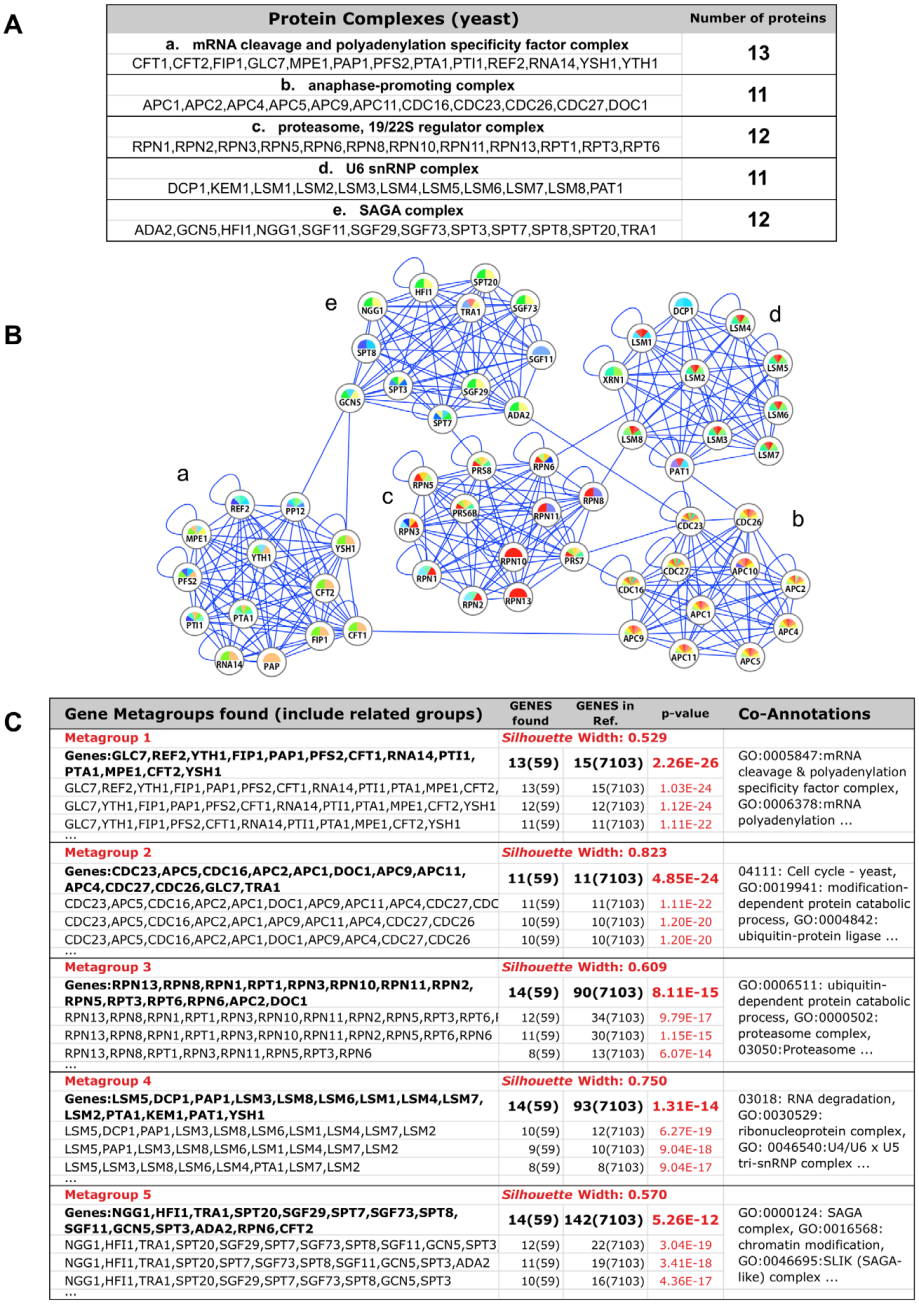
Analyses of a highly connected set of yeast proteins with *GeneTerm Linker*. Analyses of a set of 59 yeast proteins using the algorithm proposed. *(A)* Lists of the proteins that form 5 known protein complexes. *(B)* Protein interaction network form by such 59 yeast proteins. Each node is a protein and the color scheme corresponds to GO-BP and InterPro terms marked using APID2NET [17]. *(C)* Output of the analysis of the 59 genes with the algorithm proposed (full table in **Table S1**).

We investigate the ability of ***GeneTerm Linker*** method to find metagroups of functionally related genes using as test set of 59 nuclear proteins from yeast (**Figure 2A**) that have been characterized by protein interaction methods and form five well-defined protein complexes [15]. This set had been previously used in the evaluation of a method to find densely connected regions in protein interaction networks [15] and it includes a collection of well-annotated proteins with strong functional links.

A network of experimentally proven interactions between these proteins was build, using APID and APID2NET [16], [17], showing that they form 5 distinct clusters (**Figure 2B**). These clusters constitute a good set for use as a benchmark.

The analysis of the set of yeast proteins is shown in **Figure 2C**. The output of the algorithm shows that five compact metagroups are found, all having a *Silhouette Width*>0.5, that is a good indication of the internal tightness of each metagroup and its external separation from the other metagroups [14]. Moreover, the *Hypergeometric test* also indicates that the metagroups are significant. The size of the 5 metagroups found was: [1] 13 genes and 9 *GeneTerm-sets*; [2] 11 genes and 4 *GeneTerm-sets*; [3] 14 genes and 9 *GeneTerm-sets*; [4] 14 genes and 13 *GeneTerm-sets*; [5] 14 genes and 14 *GeneTerm-sets*. The terms corresponding to each metagroup are presented in **Figure 2C** (co-annotations column), showing the main functions and biological roles found associated to each metagroup (a complete version of this table is included in **Table S1**). Some concurrent terms are synonymous, like in the 3^rd^ metagroup “proteasome complex” (GO:0000502) and “proteasome” (KEGG:03050); but other terms are complementary, like in the 4^th^ metagroup “U4/U6 tri-snRNP complex” (GO:0046540) and “Like-Sm ribonucleoprotein (LSM) domain” (IPR001163). The overall result shows that the method finds the 5 complexes expected, including in each one all its proteins. In the case of metagroups 3, 4 and 5 some extra proteins are included: APC2 and DOC1 in the 3^rd^ metagroup; PAP1, PTA1 and YSH1 in the 4^th^ metagroup; and RPN6 and CFT2 in the 5^th^ metagroup.

### Comparison of the method with another functional annotation approach

To perform a comparative analysis with other methods, we carried out a systematic identification of the gene pairs that compose the test set of five yeast complexes, described above, and all the gene pairs found by the functional association method. In this way, we count all possible gene pairs and all true positive (TP) gene pairs found in the reference complexes, and we can calculate the *Accuracy* (i.e. *Rand statistic*) and the *Jaccard coefficient* defined as:
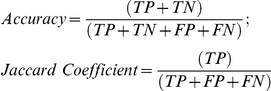



These parameters measure the relationship between pairs of points using the co-occurrence matrices for the expected partition and the partition generated by a given method [18]. The statistical evaluation was done (see **Table 1**) for the results obtained with our method and for the results obtained with a widely used *Functional Annotation Clustering* (FAC) method developed by DAVID Bioinformatics Resources [4]. This is the only method that we found in the literature that has a similar goal of finding functional modules (that include genes and terms) and use data derived from enrichment analysis.

**Table 1:** Comparison of methods: *GeneTerm Linker* and *Functional Annotation Clustering*.

	*GeneTerm Linker*	DAVID *FAC* (used by default)	DAVID *FAC* (tuned to find 5 groups)
**Total groups reference**	5	5	5
**Total groups found**	5	15	5
**All possible gene pairs**	1711	1711	1711
**TP**	320	320	254
**FN**	82	1179	132
**FP**	0	0	66
**TN**	1309	212	1259
***Jaccard Coefficient***	**0.769**	**0.213**	**0.562**
***Accuracy***	**0.952**	**0.311**	**0.884**

Comparative results for the set of 59 yeast proteins: *Accuracy* and *Jaccard Coefficient* obtained using the present method and using Functional Annotation Clustering (FAC) method with its parameters by default or tuned to find 5 groups.

The results indicate that ***GeneTerm Linker*** method is quite accurate to find the biological complexes present in the test set of 59 yeast nuclear proteins (*Accuracy* = 0.95). Such *Accuracy* drops when using the agglomeration algorithm FAC [4], which by default finds many more groups or modules of genes and terms (15 functional modules). Tuning the parameters of FAC algorithm to find just the 5 expected metagroups the *Accuracy* still does not reach 90% (0.88).

The *Jaccard coefficient* measures the proportion of gene pairs that belong to the same metagroup in both the expected and the computed partition, relative to all pairs that belong to the same metagroup in at least one of the two partitions. This *coefficient* for the case studied was 0.769 using our method and 0.562 using FAC method.

### Testing the method with reference sets from three heterogeneous resources: Complexes, Pathways and Diseases

**Figure 3:**
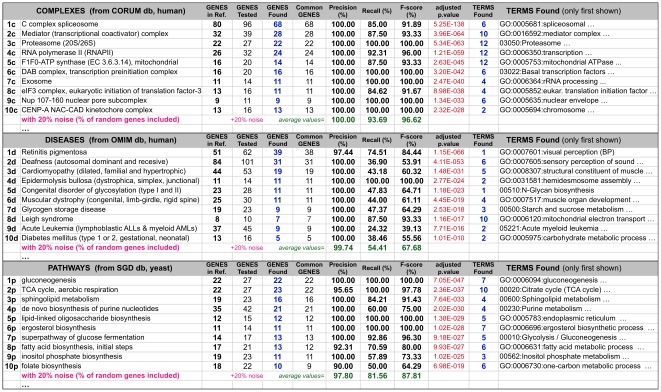
Analysis of gene sets from 3 biomolecular resources: CORUM, OMIM, SGD. Results of the analysis of thirty gene sets derived from three biomolecular resources: mammalian multiprotein complexes (CORUM), human diseases (OMIM) and yeast cellular pathways (SGD). Each row corresponds to an independent gene set and it includes the result of the functional analysis showing the first metagroup obtained running the method. Each analysis is evaluated with respect to the reference gene sets calculating the *Precision*, *Recall* and *F-score* (in %). The analyses are done introducing 20% random noise; meaning the proportion of random-selected genes added to each query gene set. The number of terms found is indicated in each row. Not all the terms are described due to space restrictions (last column). A complete table, including also the results at 60% random noise and all the information about the specific genes and terms found in each metagroup, is provided as **Table S2**.

To achieve a more comprehensive evaluation of the method, we did a series of trials with reference sets of gene metagroups defined in three broad biomolecular resources: ***(1)*** sets composed of multiprotein complexes identified in mamals (from CORUM) [19], ***(2)*** sets composed by groups of genes involved in yeast pathways (from SGD) [20], ***(3)*** sets of groups of genes involved in human diseases (from OMIM) [21]. We select from each database ten of sets with at least 8 genes/proteins each (**Figure 3**).

Using this collection of reference gene sets we run the method once for each set, to investigate how many of the reference genes are included in the first, most significant, metagroup found. We performed the analyses using not just each reference metagroup alone, but also mixing it with randomly selected genes to introduce two levels of noise in the set: 20% and 60% (i.e. in order to acquire 20% noise, if the reference group had 10 genes then 2 genes were randomly selected from the whole gene list of such resource and included with the 10 true genes).

The results using ***GeneTerm Linker*** over the whole collection of reference gene sets is shown in **Figure 3**, which presents in each row the most significant metagroup found and its overlap with the corresponding reference gene set used as query. For example, in the case of the first group (1c): the *C complex spliceosome* is composed of 80 genes, 96 genes are tested (introducing 20% extra randomly selected genes) and the method finds 68 genes, all included in the reference set and functionally linked to 6 terms with a significance of 5.25 e^−138^ (adjusted ***p-value***). Following the same steps, we calculate the results for each one of the thirty reference gene sets. As indicated above these reference sets were taken from three heterogeneous biological sources: complexes (c), diseases (d) and pathways (p). A complete table, including all the results about the specific genes and terms found in each metagroup, is provided as **Table S2**.

### Calculating the *Precision*, *Recall* and *F-score* of the method

Since the correct answer is known for each metagroup of the reference gene sets, we can calculate the error rates and estimate the *Precision* and *Recall* of our method. In an information retrieval scenario, *Precision* is defined as the number of relevant document-items retrieved by a search divided by the total number of document-items retrieved by that search, and *Recall* is defined as the number of relevant document-items retrieved by a search divided by the total number of existing relevant document-items (which should have been retrieved). The document-items in our context are the genes. The balanced *F-score* is a measure that combines *Precision* and *Recall* evenly weighted, being the harmonic mean of both. In statistical terminology these parameters –related to type I and type II errors– are defined as:
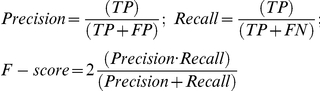



The *Precision* is a measure of exactness and fidelity, whereas the *Recall* is a measure of completeness. The results (**Figure 3**) reveal that the new functional analysis method proposed is quite precise, because it shows an average *Precision* of 100%, 99.7% and 97.8% in the identification of gene metagroups from protein complexes, diseases and pathways, respectively. Such *Precision* was obtained using a noise level of 20%. This also indicates that it is a rather robust method which allows perturbation in gene lists without losing the major functional signal included in a given metagroup.

The *Recall* –also with 20% noise– was 93.6% and 81.5% for the gene sets obtained for multiprotein complexes and pathways, respectively; and 54.4% for gene sets assigned to protein diseases. This is an interesting observation because it seems that the decrease of the *Recall* follows the same tendency expected if we were considering the strength of “functional units”. It is easy to understand that the average cohesion and tightness of the genes associated in multiprotein complexes (i.e. in “molecular machines”) should be higher that the cohesion of the genes associated within a pathway, and much stronger that the cohesion of the genes associated to a disease. In fact, many times there is not a clear functional reason about why a human gene is associated to a given disease [21]. The association is most times heuristic, observational, phenomenological, and not really linked to a known biomolecular cause. This reasoning also provides support to the method, since it shows its power to unravel different types of functional associations, and to disclose cases where the “functional units” holding the linkage between genes are not so well defined.

Finally, it seems that the size of the query groups does not affect the error rates of the method, because sets from 8 to 84 genes were assayed and the values of *Precision* and *Recall* were not dependent on the size. The only need is that each metagroup has to include a minimal number of genes to retrieve enough annotations and terms that allow functional associations. We observed that bellow seven genes it was quite difficult to achieve the linkage between genes and terms, although we do not consider it a critical constraint for high-throughput analysis.

## Discussion

### Inferring functional linkage between genes and biological terms

Some eloquent studies have asserted that *functional annotation* has become a bottleneck in biomedical science in the current era of high-throughput sequence and structure determination [22], [23]. Many genes and gene products are normally annotated by homology, assigning known functions to similar sequences. This procedure can be a potential error-prone which propagates and can contaminate most of the biomolecular databases [23]. The lack of specific knowledge about the biological function of many genes added to a recurrent annotation by simple homology and the frequent use of some terms that become “fashionable” or “promiscuous” under the influence of certain biomedical areas (e.g. cancer) can be a pitfall for many functional enrichment approaches.

**Figure 4:**
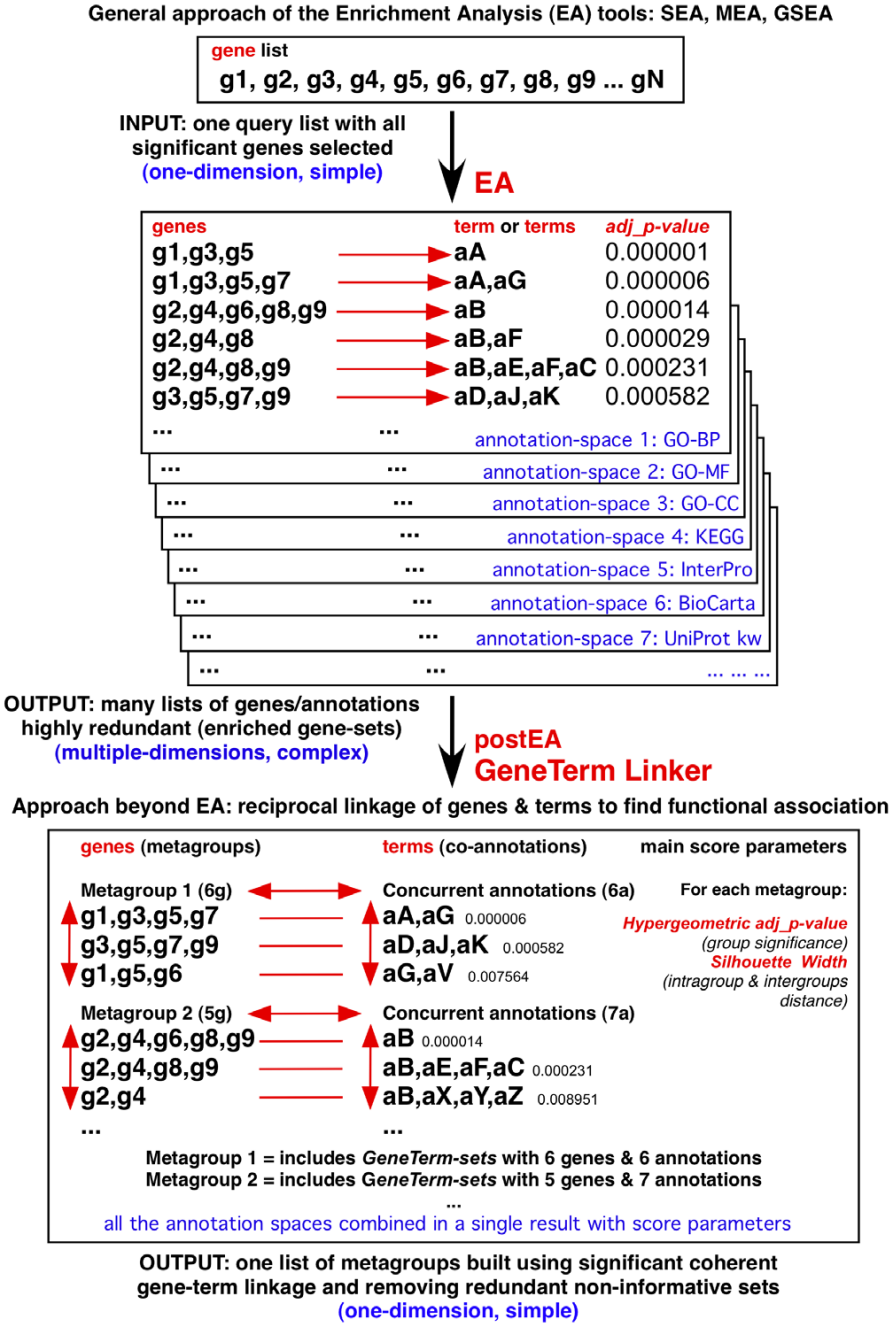
Scheme of the rational followed by *GeneTerm Linker* method. Scheme that illustrates the rational followed by the ***GeneTerm Linker*** method proposed. The method provides a single result combining all annotation spaces where a gene list has been interrogated. The method uses filters for promiscuous and redundant terms/annotations as it is described in the step 1 and 3 of the algorithm.

Using several information theory principles, we propose a new method for biological functional analysis called ***GeneTerm Linker***, developed with a clear aim of avoiding redundancy and reducing complexity in computational functional annotation, also aiming to combine multiple annotation resources. In **Figure 4** we present a scheme that illustrates the rational followed by ***GeneTerm Linker***. The power of the method is given by the fact that it combines all sources of annotations and biological information regardless of their internal structure in order to provide a single result, in this way it brings together all annotation spaces where a gene list had been interrogated. Lots of efforts have been devoted to use gene ontology (GO) as a main functional annotation space and to find functional similarity metrics in GO using its hierarchical structure and the relationship between its terms. While this is a valid approach, its application cannot be exported to other resources of non-hierarchical but very relevant biological information. As shown in **Figure 4**, our method is able to locate in the same frame terms from GO and from other annotation spaces (KEGG, InterPro, etc) providing metagroups of genes and terms linked with significance scores.

A secondary contribution of our study is to present a comparative analysis of different annotation resources. **Figure 1** reflects that KEGG annotations are more stable and contain less outliers than GO. This is caused by the existence of a thorough curation in KEGG and the fact that GO is, by definition, an ontology resource based on a controlled vocabulary, that many times has to take general broad terms applicable to genes present in very different organisms. We showed that the lack of specificity and the overuse of certain popular terms (e.g. *signal transduction* or *regulation of transcription*, **Figure 1B**) produce a strong influence on the power of the annotation resources and on the quality of their specific application to large query gene lists. Functional characterization of large gene lists, derived from genome-wide experiments, aims ideally to provide a set of annotated groups of genes that should be smaller than the number of genes in the query list [24]. However, currently most researchers in the field realize that is quite difficult to obtain a single and meaningful result using the functional enrichment tools available. The method here proposed (**Figure 4**) solves this problem providing a unique result where the related genes and terms are fuzzy enclosed in metagroups which are evaluated by enrichment, functional coherence and similarity.

In conclusion, after search and comparison with other methods, we can say that the innovation and genuine value of the algorithm presented is to provide a single coherent solution to the problem of functional annotation of lists of genes or proteins. To achieve this, it address the problem of using multiple non-orthogonal and non-homogeneous biological annotation spaces, going beyond enrichment analysis (EA) approaches that provide many lists of genes and annotations usually not integrated, redundant or with low information content. Knowing the use and value of these enrichment approaches, a clear practical problem remains for many biologists that try computer-driven exploration of their candidate gene lists. We expect that the method here presented, ***GeneTerm Linker***, will help to alleviate such difficulties offering a step forward to many gene-based biomedical and biomolecular studies.

## Materials and Methods

### Reference sets to test the method

A reference set of 59 nuclear proteins from yeast (*Saccharomyces cerevisiae*) that form five well-defined protein complexes [15] was selected as first test set and used in the comparative analysis versus the FAC method [4]. The method was also tested using 30 reference sets of gene metagroups from three biomolecular resources: ***(1)*** CORUM, comprehensive resource of mammalian multiprotein complexes [19]; ***(2)*** SGD, yeast resource that includes a collection of groups of genes involved in cellular pathways [20]; ***(3)*** OMIM, resource that includes groups of genes involved in human diseases [21]. We downloaded these 3 resources and searched for groups composed of at least 8 genes/proteins assigned to specific biological entities within each database, i.e.: assigned to specific multiprotein complexes (c), diseases (d) or pathways (p). Then, we select from each database 10 groups and consider them as reference metagroups in order to test how our method was able to find such groups. The groups are numbered 1c-10c, 1d-10d and 1p-10p. The names of the 10 groups selected from each database are included in **Figure 3** and all the details about the genes included in each reference metagroup are provided in **Table S2**.

### Formal definition of *GeneTerm-sets*

The input to the algorithm are elements defined as *GeneTerm-sets* that correspond to combinations of genes/terms/***p-value*** (considered *frequent itemset*) derived from functional annotation enrichment:
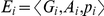

***E_i_*** *ith* element; ***G_i_*** 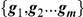 set of genes; ***A_i_*** 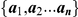 set of terms; ***p_i_ p-value***

### Mathematical description of the calculation of distances

For each element ***E_i_*** a vector ***v_i_*** contains the occurrence of each gene with respect to the whole input gene list and the ***p-value*** of each element ***E_i_*** weighted by factor ***M*** = total number of genes in the list:
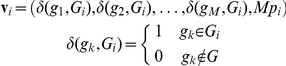
The pair-wise distances between all vectors ***v_i_*** are calculated using the *Cosine Similarity* that is derived from the *Jaccard Similarity coefficient*:
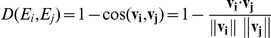



### Mathematical description of *complete cover* and application to redundancy removal

Each resulting metagroup is formed by a selected collection of *GeneTerm-sets* that keep maximum similarity. The redundancy within the preliminary metagroups is eliminated calculating the *complete cover* of each metagroup (to guarantee the completeness of the data) and then removing the *GeneTerm-sets* that do not include any new gene or any new term. Formally:
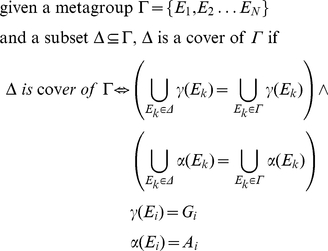



## Supporting Information

Table S1 **Complete functional analysis of 59 yeast proteins using** ***GeneTerm Linker*** **method.** Data file (.xls) containing the complete results provided by ***GeneTerm Linker*** corresponding to the functional analysis of the 59 nuclear yeast proteins (which has been partially presented in **Figure 2C**). The file has two spreadsheets: *(A)* includes a complete view of the same table as **Figure 2C**; *(B)* includes the complete output results provided by ***GeneTerm Linker*** algorithm, showing the five metagroups found with all *GeneTerm-sets* assigned to each metagroup.
(XLS)

Table S2 **Complete functional analysis of 30 gene sets from 3 resources (CORUM, OMIM and SGD) using** ***GeneTerm Linker*** **method.** Data file (.xls) containing the complete results provided by ***GeneTerm Linker*** corresponding to the analysis of 30 gene sets derived from 3 biomolecular resources: CORUM, OMIM and SGD (which has been partially presented in **Figure 3**). Each row corresponds to the functional analysis of one gene set and shows only the *first metagroup* found by the method. All genes and terms found in the *first metagroups* of each gene set are included, together with the statistical parameters (*Precision*, *Recall* and *F-score* in %) and the adjusted ***p-value*** corresponding to such metagroups. Each analysis is done twice for each gene set, introducing 20% or 60% random-selected genes.
(XLS)
